# Genetic mapping of semi-polar metabolites in pepper fruits (*Capsicum* sp.): towards unravelling the molecular regulation of flavonoid quantitative trait loci

**DOI:** 10.1007/s11032-013-9967-0

**Published:** 2013-10-23

**Authors:** Yuni Wahyuni, Vanessa Stahl-Hermes, Ana-Rosa Ballester, Ric C. H. de Vos, Roeland E. Voorrips, Awang Maharijaya, Jos Molthoff, Marcela Viquez Zamora, Enny Sudarmonowati, Ana Carolina Maisonnave Arisi, Raoul J. Bino, Arnaud G. Bovy

**Affiliations:** 1 Wageningen UR Plant Breeding, 6708 PB Wageningen, The Netherlands; 2 Research Centre for Biotechnology, Indonesian Institute of Sciences, Jl. Raya Bogor KM. 46, Cibinong, Bogor, 16910 Indonesia; 3 Present Address: Departamento de Ciência e Tecnologia de Alimentos, Centro de Ciências Agrárias, Universidade Federal de Santa Catarina, Rod. Admar Gonzaga, 1346, Florianópolis, SC 88034-001 Brazil; 4 Present Address: Instituto de Agroquímica y Tecnología de Alimentos, Consejo Superior de Investigaciones Científicas (IATA-CSIC), Avenida Agustín Escardino 7, 46980 Paterna, Valencia Spain; 5 Plant Research International, 6700 AA Wageningen, The Netherlands; 6 Netherlands Metabolomics Centre, Einsteinweg 55, 2333 CC Leiden, The Netherlands; 7 Present Address: Bogor Agricultural University, Jl. Raya Darmaga, 16680 Bogor, Indonesia; 8 Laboratory of Plant Physiology, Wageningen University, 6700 AR Wageningen, The Netherlands

**Keywords:** *Capsicum*, F2 population, Semi-polar metabolites, mQTL, Flavonoids, *MYB12*

## Abstract

Untargeted LCMS profiling of semi-polar metabolites followed by metabolite quantitative trait locus (mQTL) analysis was performed in ripe pepper fruits of 113 F2 plants derived from a cross between *Capsicum annuum* AC1979 (no. 19) and *Capsicum chinense* No. 4661 Selection (no. 18). The parental accessions were selected based on their variation in fruit morphological characteristics and fruit content of some target phytonutrients. Clear segregation of fruit colour and fruit metabolite profiles was observed in the F2 population. The F2 plants formed three clusters based on their metabolite profiles. Of the total of 542 metabolites, 52 could be annotated, including a range of flavonoids, such as flavone *C*-glycosides, flavonol *O*-glycosides and naringenin chalcone, as well as several phenylpropanoids, a capsaicin analogue, fatty acid derivatives and amino acid derivatives. Interval mapping revealed 279 mQTLs in total. Two mQTL hotspots were found on chromosome 9. These two chromosomal regions regulated the relative levels of 35 and 103 metabolites, respectively. Analysis also revealed an mQTL for a capsaicin analogue, located on chromosome 7. Confirmation of flavonoid mQTLs using a set of six flavonoid candidate gene markers and their corresponding expression data (expression QTLs) indicated the *Ca*-*MYB12* transcription factor gene on chromosome 1 and the gene encoding flavone synthase (*FS*-*2*) on chromosome 6 as likely causative genes determining the variation in naringenin chalcone and flavone *C*-glycosides, respectively, in this population. The combination of large-scale metabolite profiling and QTL analysis provided valuable insight into the genomic regions and genes important for the production of (secondary) metabolites in pepper fruit. This will impact breeding strategies aimed at optimising the content of specific metabolites in pepper fruit.

**Electronic supplementary material:**

The online version of this article (doi:10.1007/s11032-013-9967-0) contains supplementary material, which is available to authorized users.

## Introduction

Pepper (*Capsicum* spp.) is a member of the Solanaceae family, together with other important crops such as eggplant (*Solanum melongena*), tomato (*Solanum lycopersicum*) and potato (*Solanum tuberosum*). The genus *Capsicum* is categorised into 25 species and displays a wide range of genetic diversity. To introduce new traits into commercial hybrids, breeding programs based on interspecific crosses between cultivated and wild species, including *Capsicum annuum* L., *Capsicum chinense* Jacq., *Capsicum frutescens* L., *Capsicum baccatum* L. and *Capsicum pubescens* Ruiz and Pav. are underway. Such crosses produce fertile and heterogeneous progenies (Djian-Caporilano et al. 2007) and give opportunities to introduce economically valuable traits from wild species into the cultivated varieties. One such opportunity is the enrichment of pepper fruits with phytonutrients that encompass potential health-promoting properties.

Metabolite studies in pepper fruits have been mainly focused on the targeted analyses of specific groups of well-known pepper phytonutrients, including ascorbic acid (vitamin C), tocopherols (vitamin E), carotenoids (provitamin A), flavonoids and capsaicinoids (Wahyuni et al. 2011; Howard and Wildman 2007). Untargeted metabolite profiling techniques, such as those based on mass spectrometry (MS), allow the detection of metabolites in a biological sample, without a priori knowledge of the identity of the metabolites detected. Such a profiling approach gives the opportunity to analyse hundreds of metabolites simultaneously and provides the necessary information to elucidate metabolic relationships at the biochemical level. For instance, untargeted profiling approaches have been used to obtain an overview of the metabolic diversity in germplasm collections of *Arabidopsis* (Keurentjes et al. 2006), tomato (Tikunov et al. 2005) and pepper (Wahyuni et al. 2013), and have helped scientists to understand the genetic control of the metabolic pathways underlying the main biochemical contrasts between the genotypes under study. Metabolic pathways are generally controlled by multiple genes encoding specific biosynthetic enzymes as well as regulatory factors. The dramatic effect that both structural and transcription factor genes can have on the flux through a metabolic pathway has been demonstrated in, for example, transgenic tomatoes that produce high amounts of flavonols due to overexpression of the petunia *CHI* gene, which encodes the rate-limiting enzyme in the flavonoid pathway in tomato (Muir et al. 2001), or as the result of the introduction of transcription factor genes that up-regulate the endogenous flavonoid pathway genes in the fruit (Bovy et al. 2002; Butelli et al. 2008). The combination of metabolic and genetic analyses helps to identify genetic markers and key genes that underlie so-called metabolite quantitative trait loci (mQTLs), genomic regions which are associated with increased (or decreased) metabolite levels. Using targeted metabolite analyses approaches, the genetic regulation of specific metabolites, such as chlorophyll (Brand et al. 2012) and the pungent capsaicinoids (Ben-Chaim et al. 2006; Blum et al. 2002, 2003), has been analysed in pepper fruit. Two major QTLs, *pc8.1* and *pc10.1*, that control chlorophyll content were identified in a population based on a cross between a dark green *C. annuum* and a light green *C. chinense* accession (Brand et al. 2012). In addition, the QTL *pc8.1* also affected carotenoid content in ripe fruit in that population. In pungent peppers, the ability to produce capsaicinoids is determined by the presence of a functional *Pun1* gene on chromosome 2, which encodes the acyltransferase AT3 required for the formation of capsaicin from its aromatic precursor vanillylamine and various acyl moieties derived from the catabolism of branched-chain amino acids (Mazourek et al. 2009). A loss-of-function allele of this gene, *pun1*-*1*, is present in all commercial non-pungent pepper varieties. Up to six QTLs affecting the capsaicinoid levels in pungent accessions have been identified on chromosomes 3, 4 and 7 (Ben-Chaim et al. 2006; Blum et al. 2003).

In order to find novel mQTLs in pepper fruit, we analysed a segregating F2 population derived from a cross between the wild pepper accession *C. annuum* AC1979 (no. 19) and the cultivated accession *C. chinense* No. 4661 Selection (no. 18) for variation in semi-polar metabolites, using accurate mass LC-QTOF-MS. Subsequently we carried out an mQTL analysis using amplified fragment length polymorphism (AFLP) and microsatellite marker data available for this population (Maharijaya et al. 2012b). The parental accessions were selected based on the results of a previous study with 32 pepper accessions (Wahyuni et al. 2011, 2013) and differed in several metabolic traits: *C. annuum* AC1979 (no. 19) is highly pungent and has a high level of capsianosides (diterpene glycosides) and luteolin-*O*-glycosides (Wahyuni et al. 2011), whereas *C. chinense* No. 4661 Selection (no. 18) showed high levels of the flavonoid naringenin chalcone (Wahyuni et al. 2013). The two accessions also differed for their susceptibility to two thrips species, *Frankliniella occidentalis* and *Thrips parvispinus*: leaves of *C. annuum* AC1979 (no. 19) showed a high thrips resistance, while *C. chinense* No. 4661 Selection (no. 18) was very susceptible (Maharijaya et al. 2011, 2012a). This approach revealed valuable insight into the genomic regions important for the production of (secondary) metabolites in pepper fruit. Confirmation of the results using a candidate gene approach, in which flavonoid mQTL, gene expression (eQTL) and candidate gene marker data were combined, indicated several flavonoid genes as the causative gene underlying important flavonoid QTLs and provided valuable insight into the molecular regulation of the flavonoid pathway in pepper fruit. The impact of this approach on future breeding strategies aimed at developing new pepper varieties with a desired composition of specific metabolites will be discussed.

## Materials and methods

### Plant materials

An interspecific F2 population was derived from a cross between *C. annuum* AC1979 (no. 19) as the female and *C. chinense* No. 4661 Selection (no. 18) as the male (Supplemental Fig. 1), previously described by Maharijaya et al. (2012b). In short, seeds of the two parental accessions were obtained from the Centre for Genetic Resources, The Netherlands (CGN). After crossing the two accessions, the F1 plants were grown in a controlled environmental greenhouse located in Wageningen (The Netherlands) from December 2007 until September 2008. Two F1 plants showing homogeneous phenotypes were chosen and were self-pollinated to obtain the F2 generation. The F2 population was subsequently cultivated from December 2008 until September 2009 and consisted of 201 plants: 81 plants from F1-1 and 120 plants from F1-2. These plants were randomly arranged in eight rows of 32 plants: including F2 plants, cuttings of F1 plants and the parental plants. Of the 201 F2 plants, only 113 plants produced enough fruits for further analysis and, depending on fruit size, 10–50 ripe fruits were harvested from each of these plants. Whole fruits were frozen in liquid nitrogen, ground and stored at −80 °C for further metabolite analysis.

### Extraction and analysis of semi-polar metabolites

Semi-polar metabolites were analysed from whole ripe fruits of 113 F2 plants, two F1 plants, and the parents *C. annuum* AC1979 (no. 19) and *C. chinense* No. 4661 Selection (no. 18). Metabolite extraction was performed according to de Vos et al. (2007). Briefly, ripe pepper fruits were frozen in liquid nitrogen and ground to a powder. Aliquots of 500 mg frozen powder were extracted with 1.5 ml of 99.875 % methanol acidified with 0.125 % formic acid. The extracts were sonicated, centrifuged and filtered through a 0.2-μm polytetrafluoroethylene (PTFE) filter. To check for total technical variations (i.e. extraction, sample analysis and data processing), quality control samples were prepared by pooling fruit material that was extracted 10 times using the same procedure and injected after every 15 extracts.

All extracts were analysed using reversed phase liquid chromatography (Waters Alliance HPLC 2695) coupled to a photodiode array detector and a quadrupole time of flight high-resolution mass spectrometry (LC-PDA-QTOF-MS) system (Waters QTOF-Ultima), using C18-reversed phase chromatography (Phenomenex C-18 Luna column) and negative electrospray ionisation, mainly as described previously (de Vos et al. 2007). Extracts were diluted 10 times to prevent ionisation saturation and 5 μl of the diluted extract was injected and separated using a binary gradient of ultrapure water (A) and acetonitrile (B), both acidified with 0.1 % formic acid, with a flow rate of 0.19 ml/min. The initial solvent composition consisted of 95 % of A and 5 % of B, increased linearly to 35 % A and 65 % B in 45 min and maintained for 2 min. The column was washed with 25 % A and 75 % B for 5 min and equilibrated to 95 % A and 5 % B for 2 min before the next injection.

### Metabolite data processing

Semi-polar metabolite data were processed as described below:

#### Mass spectral alignment, filtering and clustering

The dataset was processed using the MetAlign software package (www.metalign.nl) for baseline correction, noise estimation and ion-wise mass spectral alignment (Lommen and Kools 2012). Absent values were replaced by the local noise. The MetAlign output was processed with MSClust for data reduction and extraction of compound mass spectra through clustering of individual ions originating from the same metabolite (Tikunov et al. 2012). A total of 542 so-called reconstructed metabolites were thus obtained and used for mQTL analyses.

#### Putative identification of semi-polar metabolites

The identification of semi-polar metabolites was based on their UV spectra (if present), observed accurate mass (LC-QTOF-MS) and MS^n^ fragmentation patterns (LTQ-Orbitrap FTMS), as described by Wahyuni et al. (2013). Putative identification of semi-polar metabolites was obtained using different metabolite databases such as Dictionary of Natural Products (http://dnp.chemnetbase.com) and KNApSAcK (http://kanaya.naist.jp/KNApSAcK) and based on comparison with an accurate mass in-house metabolite database developed from literature data (Marín et al. 2004) and previous metabolomics experiments in combination with accurate MS^n^ fragmentation experiments (Wahyuni et al. 2011, 2013).

#### Multivariate analysis

Semi-polar metabolite datasets containing the intensity levels of all centrotypes for all pepper samples were analysed separately using multivariate statistical analyses included in the Genemath XT version 1.6.1 software. Pre-treatment of the data was performed by log_2_ transformation and mean centering. 542 metabolites were subjected to principal component analysis (PCA) and hierarchical cluster analysis (HCA). HCA was performed by using the UPGMA method and Pearson’s coefficient matrix in Genemath XT software. To test the reliability of the dendrogram produced by HCA, bootstrap analysis was performed with 100 replications.

### Genetic linkage map construction and metabolic QTL mapping analysis

A genetic linkage map was constructed with AFLP and microsatellite markers as described by Maharijaya et al. (2012b). In brief, 15 primer combinations were used to develop the following AFLP markers: P17-M39, P17-M32, P14-M50, P14-M49, P14-M48, P14-M41, P11-M61, P11-M48, E38-M49, E36-M48, E35-M58, E35-M49, E35-M48, E34-M48 and E32-M49. The sequence of these primers followed the standard list for AFLP primer nomenclature (http://wheat.pw.usda.gov/ggpages/keygeneAFLPs.html). For microsatellite marker amplification, 56 simple sequence repeat (SSR) primers were used and the primer sequences were described in Maharijaya et al. (2012b). The microsatellite markers were used to assign linkage groups to known pepper markers (Wu et al. 2009; Yi et al. 2006; Lee et al. 2009). Based on all markers a linkage map was constructed using JoinMap 4.1 (van Ooijen 2006).

Metabolite QTL mapping analysis was performed using MapQTL 6.0 (van Ooijen 2009). Briefly, the dataset containing the relative intensities of 542 metabolites was subjected to QTL analysis. To define the significance threshold of logarithm of odds (LOD), 1,000 permutation tests (*P* ≤ 0.05) were performed on several metabolites having low LOD score values for QTL detection. This led to a LOD threshold value of 3.7.

### RNA extraction

Total RNA was extracted from 50 mg freeze-ground pericarp of ripe fruits of 113 F2 segregating plants and the two parents *C. annuum* AC1979 (no. 19) and *C. chinense* No. 4661 Selection (no. 18) using RNeasy Plant Minikit (Qiagen) according to the manufacturer’s instructions. The quality and quantity of total RNA were measured by a Nanodrop spectrophotometer ND1000 (Isogen Life Science) and evaluated by electrophoresis using 200 ng of the total RNA from each sample on 1.2 % w/v agarose gel. cDNA was synthesised from 1 μg of total RNA using Taqman^®^ Reverse Transcription (Applied Biosystems) according to the manufacturer’s instructions.

### Selection and isolation of pepper flavonoid candidate genes

Putative flavonoid candidate gene sequences were selected from the pepper gene index database (http://compbio.dfci.harvard.edu/cgi-bin/tgi/gimain.pl?gudb=pepper), based on homology with known flavonoid genes from tomato, i.e. *chalcone synthase* (*CHS*), *chalcone isomerase* (*CHI*), *flavanone 3*-*hydroxylse* (*F3H*)*, flavonol synthase* (*FLS*), and *flavonoid 3′*-*hydroxylase* (*F3′H*). In short, the tomato genes were compared with the pepper gene index using BLAST-N and the five best hits for each gene were re-BLASTed against the tomato genome. Only those expressed sequence tags showing their best hit with a known tomato flavonoid gene were considered as a candidate for gene expression and/or marker analysis. In addition, a candidate *flavone synthase*-*2* (*FS*-*2*) was selected based on homology with the *Lobelia erinus* *FS*-*2* gene. Furthermore, a *MYB12* transcription factor gene (coded as *Ca*-*MYB12* in this report) was isolated from pepper cDNA using the rapid amplification of cDNA ends (RACE)-PCR technique, using primer sets derived from the *SlMYB12* gene. For each candidate gene, a set of primers was designed using Primer3Plus (http://probes.pw.usda.gov/cgi-bin/batchprimer3/batchprimer3.cgi). Candidate genes and primer sequences are described in Supplemental Table 1.

### Flavonoid gene expression analysis

Gene expression analysis was carried out by quantitative real-time PCR (qRT-PCR) using an iCycler iQ machine (Bio-Rad Laboratories) with iQ SYBR Green Supermix (Bio-Rad Laboratories) as fluorescent dye. The housekeeping gene *α*-*tubulin* was chosen to normalise the expression levels of candidate genes, due to the stability of its Ct (threshold cycle) value over all samples (results not shown). Expression levels of candidate genes were determined by the delta Ct (ΔCt) values, calculated by subtracting the Ct value of each candidate gene from the Ct value of *α*-*tubulin*. For easy representation, 2^−ΔCt^ was calculated, multiplied by 100 and converted to log_2_, as described by Livak and Schmittgen (2001).

### Detection of SNPs in flavonoid candidate genes and mapping of the genetic position of flavonoid SNP markers

Single nucleotide polymorphism (SNP) detection was carried out by sequence analysis of PCR fragments of 14 flavonoid genes and one *R2R3*-*MYB12* transcription factor gene obtained from cDNA and/or genomic DNA of the two parents of the F2 population. Based on SNPs detected on sequences from both parents, a pair of specific primers was designed using Primer-Picker (KBioscience, UK) for each SNP (Supplemental Table 1) and genotyping of the F2 population was carried out using KASPar SNP Genotyping (KBioscience, UK). A subset of SNP profiles was integrated with AFLP and microsatellite markers to reconstruct the genetic map using JoinMap 4.1 (van Ooijen 2006).

### Flavonoid QTL and flavonoid eQTL analyses

Flavonoid QTLs obtained in previous analysis of this research were re-analysed with the genetic map improved with flavonoid SNP markers using MapQTL 6.0 (van Ooijen 2009). Subsequently, flavonoid gene transcript QTL (eQTL) analysis was performed with the same genetic map and software as those used to re-analysed flavonoid QTLs. The significant LOD thresholds were defined with a permutation test (1,000; *P* ≤ 0.05) on genes and several metabolites having low LOD score values for QTL detection, leading to a LOD threshold value for eQTL of 3.6 and mQTL of 3.7.

## Results

### Phenotype of parental, F1 and F2 plants

The two parental accessions that were used for generating the F2 population (Supplemental Fig. 1), *C. annuum* AC1979 (no. 19) and *C. chinense* No. 4661 Selection (no. 18), varied in fruit colour, shape and size. Fruits of *C. annuum* AC 1979 (no. 19) were small (1.5 cm) and the colour of ripe-stage fruits was red (Supplemental Fig. 2a), while *C. chinense* No. 4661 Selection (no. 18) produced large (8.5 cm) brown ripe fruits (Supplemental Fig. 2b). Ripe fruits of F1 plants were red [similar to the female parent *C. annuum* AC1979 (no. 19)], with a length of about 2.5 cm (Supplemental Fig. 2c). The ripe fruit colour of the F2 plants segregated among individuals and ranged over dark red, red, brown and dark brown with a frequency of 20, 56, 13 and 11 %, respectively (Supplemental Fig. 2d). In addition, the fruit size segregated among F2 plants: the smallest fruit was 1 cm and the biggest reached up to 5 cm (Supplemental Fig. 2d).

### Semi-polar metabolites in ripe fruits of parental, F1 and F2 plants

**Fig. 1:**
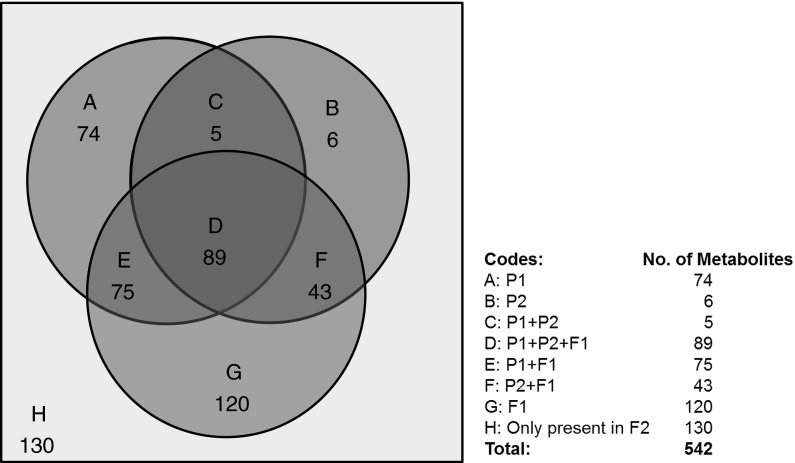
Venn diagram of 542 semi-polar metabolites detected in ripe fruits of two parental accessions, *P1* = *C. annuum* AC1979 (no. 19) and *P2* = *C. chinense* No. 4661 Selection (no. 18), and F1 and F2 plants. Values in *circles* and *intersections* illustrate the number of metabolites accumulating in each of the sample groups. A metabolite was denoted as “detected” in a given sample when the mass intensity level of its centrotype mass peak was above the local noise. According to the Metalign output, the local noise was set at a mass intensity of 37

From previous work we observed that *C. annuum* AC1979 (no. 19) accumulated relatively high levels of capsianosides, capsaicinoids, chlorogenic acid and luteolin *O*-glycosides in its fruits, while *C. chinense* No. 4661 Selection (no. 18) fruits showed relatively high levels of naringenin chalcone and several specific flavonoid glycosides, such as a flavanone-tri-methyl-hexose-pentose and quercetin 3-*O*-rhamnoside-7-*O*-glucoside (Wahyuni et al. 2011, 2013). Using untargeted LC-PDA-QTOF-MS we determined the metabolite profiles of ripe fruits from parental, F1 and F2 plants. In total, 542 semi-polar metabolites were detected over all samples, of which 74 metabolites (14 %) were detected only in parent *C. annuum* AC1979 (no. 19) and six metabolites (1 %) were detected only in *C. chinense* No. 4661 Selection (no. 18) (Fig. 1; circles A and B). Despite those differences, the two parents also shared five metabolites (0.9 %) which were not detected in the two F1 plants (Fig. 1; intersection C). The fruit metabolite profiles of the two F1 plants showed that these plants were identical and contained 120 metabolites (22 %) that were below detection limits in either parent (Fig. 1; circle G). In addition, the contribution of each parent to the metabolite profiles of the F1 was shown by the presence of 75 metabolites (14 %) derived from female parent no. 19 (Fig. 1; intersection E) and 43 (8 %) derived from male parent no. 18 (Fig. 1; intersection F). Another 89 metabolites (16 %) were shared between all F1 plants and both parents (Fig. 1; intersection D). The other 130 metabolites (24 %) were detected neither in parents nor in F1 plants, thus they were uniquely detected in fruits from F2 plants (Fig. 1; H).

**Fig. 2:**
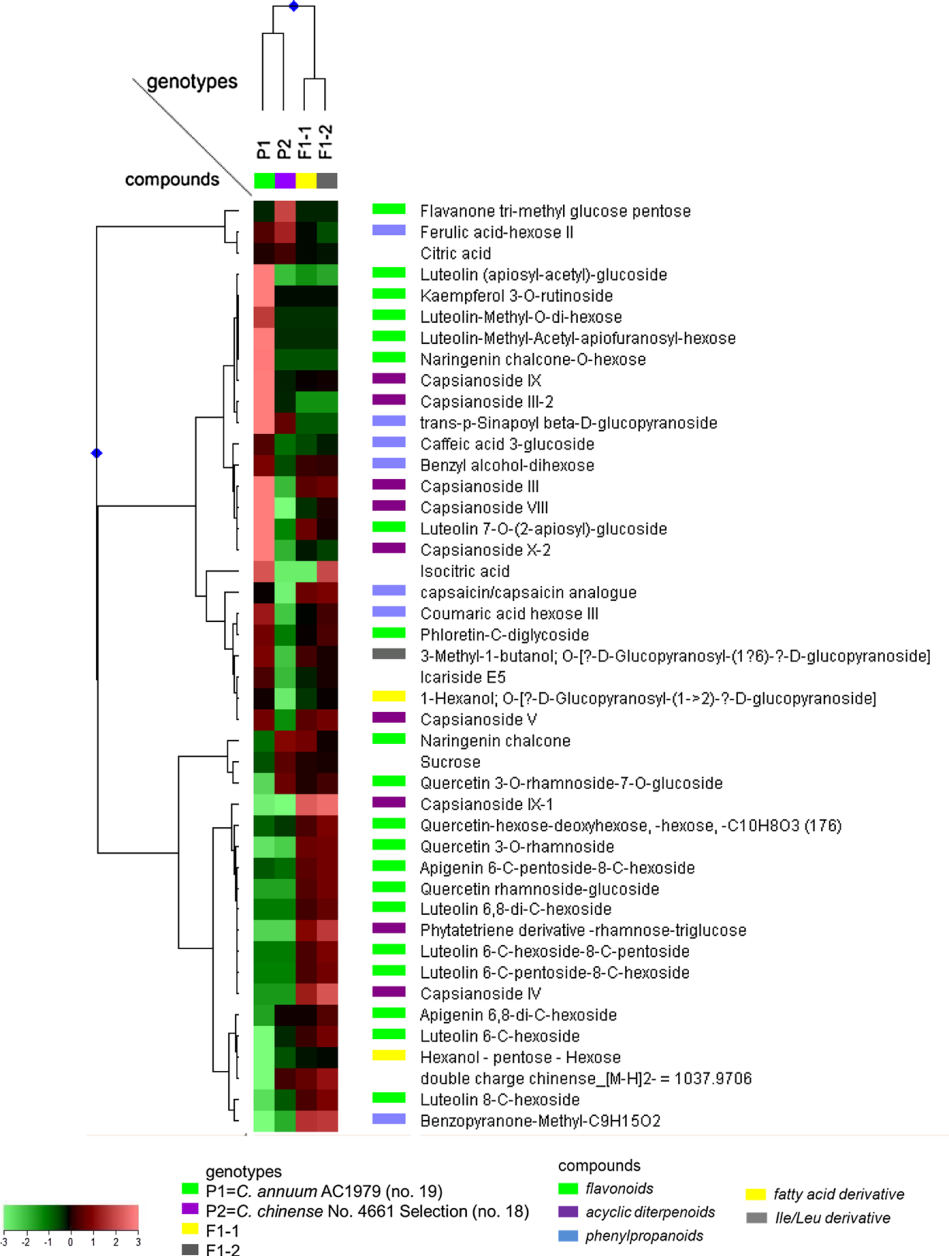
Heat map of 43 putative semi-polar metabolites which accumulated in ripe fruits of the parental accessions, *C. annuum* AC1979 (no. 19) and *C. chinense* No. 4661 Selection (no. 18), and/or the two F1 plants. A *color*-*coded matrix* represents values of the metabolite intensity in each genotype, which has been log_2_-transformed and mean-centered. (Color figure online)

Out of the 542 metabolites, 52 could be putatively identified based on comparison with an accurate mass in-house metabolite database developed from literature data (Marín et al. 2004) and from previous metabolomics experiments using the same LCMS system, in combination with accurate MS^n^ fragmentation experiments (Wahyuni et al. 2013; Supplemental Table 2). These metabolites belonged to a number of compound classes, such as flavonoids, phenylpropanoids, acyclic diterpenoids and fatty acid derivatives. Of all putatively identified metabolites, 43 accumulated at detectable levels in fruits of one or both parents and/or the F1 plants (Fig. 2), whereas 9 metabolites were uniquely found in F2 plants only. Acyclic diterpenoids with different sugar decorations, a capsaicin analogue, a lignan, fatty acid derivatives and amino acid derivatives, as well as the flavone luteolin 7-*O*-(2-apiosyl)-glucoside, were found in the female parent and F1 plants. In addition, we detected naringenin chalcone, quercetin 3-*O*-rhamnoside-7-*O*-glucoside, luteolin *C*-glycosides and some yet unknown metabolites in the male parent and F1 plants. Flavonoids, including apigenin, luteolin and quercetin glycosides were relatively abundant in F1 plants compared to the parents.

**Fig. 3:**
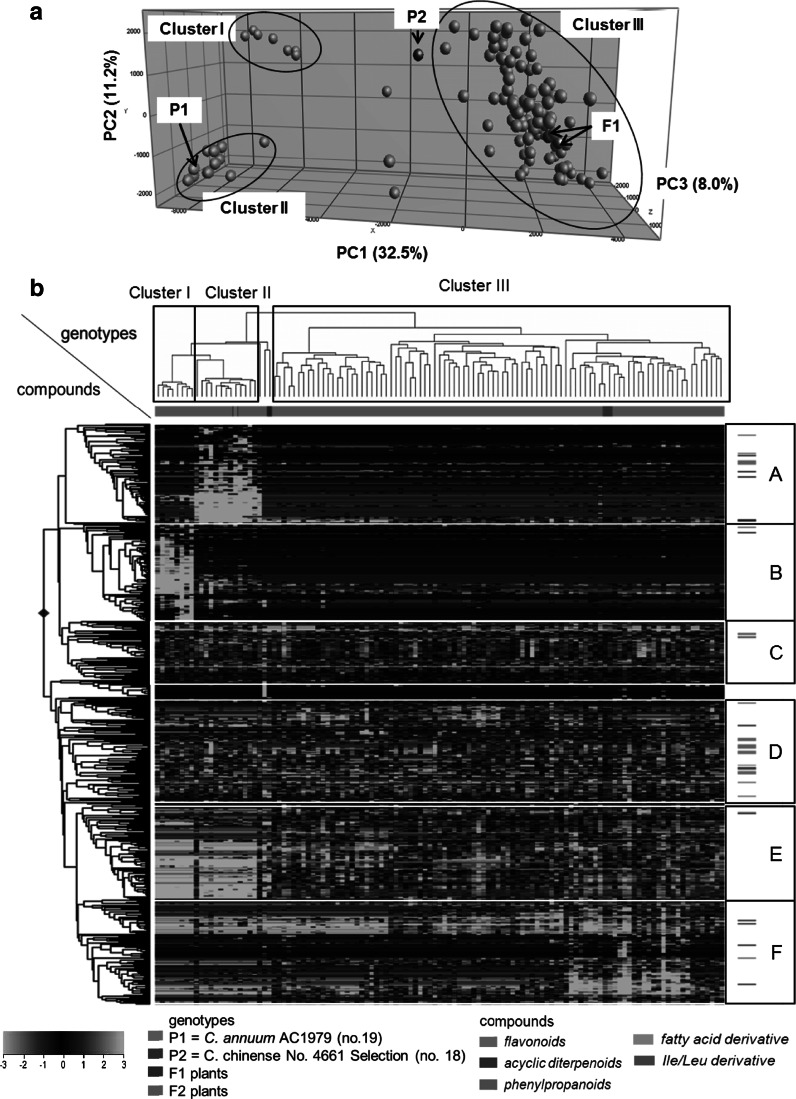
Principal component and hierarchical cluster analysis of F2 plants, two F1 plants and two parental accessions. **a** Principal component analysis. *P1* = *C. annuum* AC1979 (no. 19) and *P2* = *C. chinense* No. 4661 Selection (no. 18), based on 542 semi-polar metabolite profiles in ripe fruits. **b** Hierarchical cluster analysis. Heat map of 542 metabolites in ripe fruits of F2, two F1 and two parental accession plants, *C. annuum* AC1979 (no. 19) and *C. chinense* No. 4661 Selection (no. 18). A *color*-*coded matrix* represents the value of the metabolite intensity in pepper samples, which has been log_2_-transformed and mean-centered. The alphabets (*A*–*F*) represent metabolite clusters. Characteristics of the underlying metabolites are presented in Supplemental Table 2. (Color figure online)

The semi-polar metabolite profiles in ripe fruits of all F2 plants were subjected to PCA and HCA. Based on the variation in semi-polar metabolite profiles, PCA revealed a separation of the samples into three main clusters: Cluster I contained eight F2 plants, Cluster II consisted of 11 F2 plants plus the accession *C. annuum* AC1979 (no. 19) and Cluster III contained 92 F2 plants together with the F1 plants (Fig. 3a). Three F2 plants and the male parent *C. chinense* No. 4661 Selection (no. 18) were not assigned to a specific cluster and they showed an intermediate behaviour. The first principal component (PC1) explained 32.5 % of the variation and separated Cluster I and Cluster II from Cluster III. The second principal component (PC2) explained 11.2 % of the variation and separated Cluster I from Cluster II.

HCA of the set of 542 semi-polar metabolites revealed the presence of specific metabolite groups, arbitrarily denoted A to F, as characterised by their specific accumulation pattern across the genotypes (Fig. 3b). Structurally related metabolites derived from the same metabolic pathway may cluster together, as shown previously for volatiles in tomato (Tikunov et al. 2005) and pepper (Eggink et al. 2012; Wahyuni et al. 2013). The heat map of the 542 metabolites shows a group of 98 metabolites in group D, which contained all flavone *C*-glycosides next to flavonol *O*-glycosides, naringenin chalcone and its glycoside derivative, phenylpropanoids, a capsaicin analogue, fatty acid derivatives and amino acid derivatives (Fig. 3b; Supplemental Table 2). The metabolites in this group and all metabolites in group C were present at different relative abundances in all plants tested and their segregation pattern in the F2 plants was not specific for any of the three genotype clusters, since genotypes with high and low abundance of these metabolites were present in each cluster. Metabolites in group B accumulated at relatively high abundance in the ripe fruits from the F2 plants of Cluster I only. Three out of the 90 metabolites of group B were putatively identified as capsicosides, a group of steroidal glycosides detected previously in *C. annuum* var. *acuminatum* seeds (Yahara et al. 1994; Iorizzi et al. 2002). These compounds were reported to have antimicrobial activity against yeast and fungi (Iorizzi et al. 2002). All 94 metabolites in group A accumulated at relatively high levels in Cluster II which contained several F2 plants and the female parent (no. 19). Group A contained five capsianosides and six flavonoid *O*-glycosides, including several luteolin *O*-glycosides as well as quercetin and kaempferol *O*-glycosides. These compounds were previously shown to accumulate at relatively high levels in female parent no. 19 (Wahyuni et al. 2013) and in the F1 plants (Fig. 3b; Supplemental Table 2), in comparison to parent no. 18. The 187 metabolites in groups E and F were detected at low levels in genotype Clusters I and II and contributed to the characteristic metabolite pattern of genotype Cluster III. Metabolites of group E accumulated at relatively high levels in all plants of genotype Cluster III and contained several unique but as yet unknown metabolites, detected as doubly charged negative ions, as well as one capsianoside and one capsoside (Fig. 3b; Supplemental Table 2). Capsoside is a cell wall galactolipid that has been previously detected in very small amounts in ripe fruits of *C. annuum* var. *acuminatum* (Iorizzi et al. 2001). Other capsianosides with different glycoside decorations were present in groups D, E and F. In addition, one specific flavonol glycoside, present in group F, was detected at relatively high abundance in several F2 plants of Cluster III.

### QTL analyses of the F2 population

**Fig. 4:**
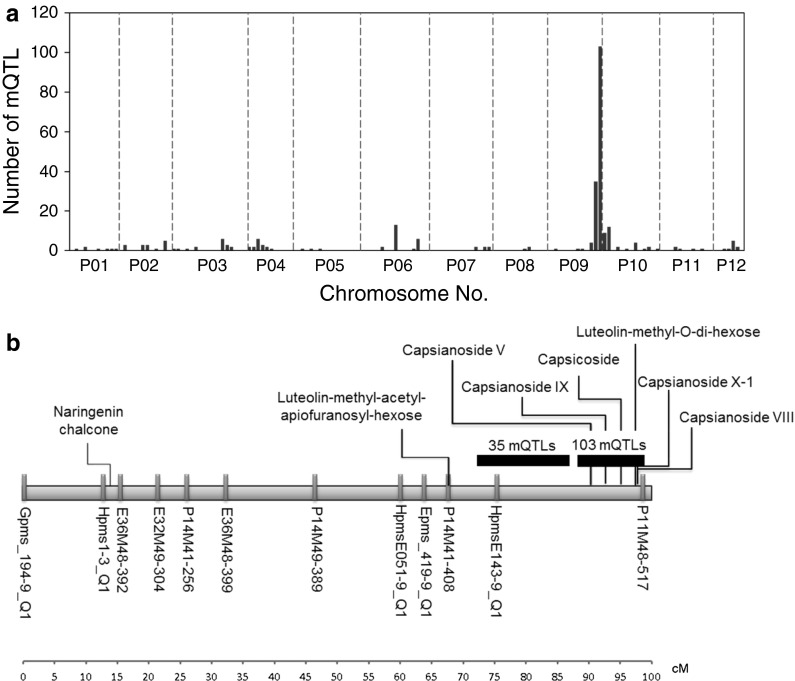
Overview of mQTLs in pepper fruit. **a** Frequency distribution of total mQTLs detected at each marker position on pepper chromosomes, represented by *red bars*. The *dotted vertical lines* represent the chromosomal borders. **b** mQTLs likelihood map on chromosome 9. (Color figure online)

To uncover genetic loci controlling the observed variation in metabolic profiles, we subsequently analysed the genetic distribution of the 542 metabolite patterns using QTL analysis. We could significantly link 231 metabolites to one or more genetic markers (based on interval mapping with a LOD threshold of 3.7), accounting for a total of 279 mQTLs. In total 24 out of these 231 metabolites were putatively annotated as belonging to several metabolite classes, including acyclic diterpenoids/capsianosides, phenylpropanoids, fatty acid derivatives, amino acid derivatives and flavonoids (Supplemental Table 4). In addition, we found several mQTLs for as yet unknown, doubly charged compounds (Supplemental Table 4). The 279 mQTLs were not evenly distributed over the genome and in particular two major QTL hotspots were detected on chromosome 9 (Fig. 4a): one mQTL hotspot harbouring 35 mQTLs co-localised with the marker HpmsE143-9_Q1 at 76 cM and a second hotspot harbouring 103 mQTLs co-localised with the marker P11M48-517 at 100 cM (Fig. 4b). The first group included several doubly charged compounds, which were detected at significant levels in both the male parent (no. 18) and the F1 plants (Supplemental Table 2). The mQTL hotspot adjacent to marker P11M48-517 included seven putatively annotated metabolites, of which five were putatively identified as capsianosides. The QTL for these capsianosides was derived from the female parent (no. 19), as demonstrated by the positive effect of the homozygous A allele on their accumulation (Supplemental Table 4) and their relative abundance in the female parent (Fig. 2). In addition, we revealed 11 mQTLs for flavonoid glycosides distributed over several genomic locations (Supplemental Table 4). QTLs for quercetin, luteolin and apigenin glycosides were located at different positions on different chromosomes, i.e. we found a QTL for quercetin rhamnoside-glucoside on chromosome 2 at 144 cM and a QTL for quercetin 3-*O*-rhamnoside on chromosome 10 at 7 cM (linkage group 10b). On chromosome 6, several QTLs for flavone *C*-glycosides, including two apigenin *C*-glycosides and two luteolin *C*-glycosides, were found in the region from 160 to 167 cM. In addition, QTLs for two methylated luteolin glycosides were found on chromosome 9 at 68 and 97 cM, in the QTL hotspot region. On the top of chromosome 9 we also found a QTL for naringenin chalcone (Fig. 4b). Furthermore, two QTLs for flavanone-3-methylglucose pentose were found on chromosome 6 and 7, respectively. QTLs for other phenylpropanoids and acyclic diterpenoids were observed at different linkage groups (Supplemental Table 4). The capsaicin analogue, which has been shown previously to be highly correlated with total capsaicinoid levels (Wahyuni et al. 2011), was linked to a QTL on chromosome 7 (98 cM).

**Fig. 5:**
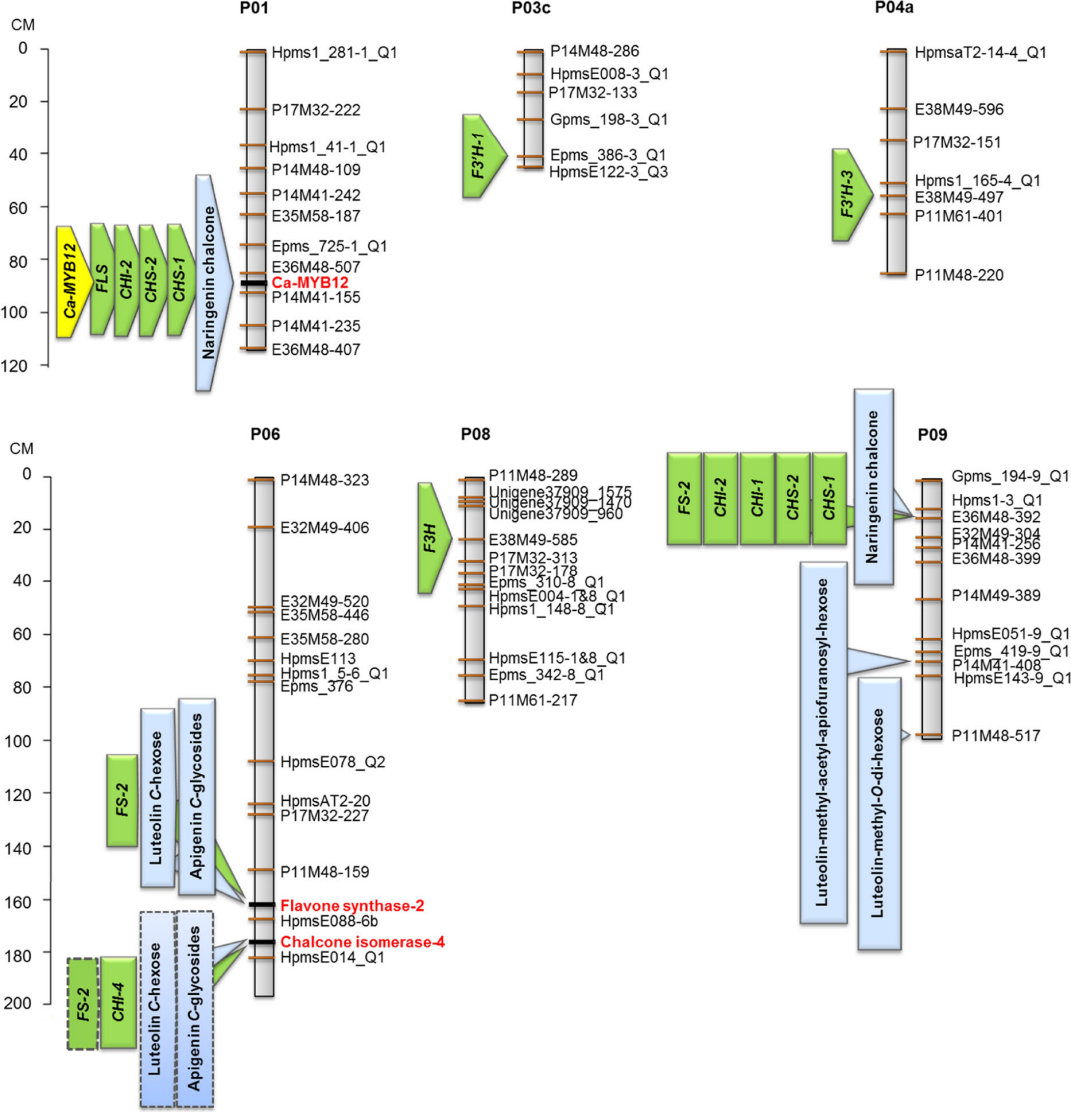
Flavonoid mQTLs and flavonoid candidate gene eQTLs on pepper chromosomes. *Light blue boxes* indicate flavonoid mQTLs, the *yellow box* indicates the *Ca*-*MYB12* transcription factor eQTL and *green boxes* indicate eQTLs of flavonoid structural candidate genes. *Boxes with grey dotted outlines* indicate flavone *C*-glycoside mQTLs and an *FS*-*2* eQTL with significant LOD scores (mQTL ≥ 3.7; eQTL ≥ 3.6) at the CHI-4 locus, but these are not considered as a separate locus for those metabolites, since they overlap with the *FS*-*2* QTL interval. *CHS* chalcone synthase, *CHI* chalcone isomerase, *F3H* flavanone-3-hydroxylase, *FLS* flavonol synthase, *F3′H* flavonoid-3*′*-hydroxylase, *FS* flavone synthase. (Color figure online)

To validate the flavonoid QTLs detected using the untargeted metabolomics approach and to gain insight in the underlying molecular regulation, three experiments were conducted: (1) mapping the genetic position of flavonoid candidate genes, (2) re-analysis of the flavonoid mQTLs using the flavonoid candidate genes as markers and (3) expression QTL (eQTL) analysis using flavonoid gene expression data. Firstly, a *Ca*-*MYB12* transcription factor gene, three *chalcone synthase* (*CHS*-*A*, *CHS*-*1* and *CHS*-*2*), four *chalcone* *isomerase* (*CHI*-*1*, *CHI*-*2*, *CHI*-*3* and *CHI*-*4*), a *flavanone*-*3*-*hydroxylase* (*F3H*), a *flavonol synthase* (*FLS*) and three *flavanone*-*3′*-*hydroxylase* (*F3′H*-*1, F3′H*-*2 and F3′H*-*3*) candidate genes were identified based on homology with known tomato flavonoid genes. In addition, a *flavone synthase* candidate gene (*FS*-*2*) was identified based on homology with the *Lobelia erinus* *flavone synthase* (*FS*-*2*) gene. For ten genes (*Ca*-*MYB12*, *CHS*-*2*, *CHI*-*1*, *CHI*-*2*, *CHI*-*4*, *F3H*, *F3′H*-*1*, *F3′H*-*3*, *FLS* and *FS*-*2*), SNPs between the two population parents were obtained and used to genotype the segregating F2 population. These data were used to locate the position of the candidate genes on the pepper genetic map. Out of these ten candidate genes, five genes could be located on chromosome regions, i.e. *Ca*-*MYB12* at 89.6 cM on chromosome 1, *CHS*-*2* on the top (0.0 cM) of chromosome 5 (linkage group 5a), *CHI*-*2* at 15.4 cM on chromosome 5 (linkage group 5a), *FS*-*2* at 162.1 cM on chromosome 6 and *CHI*-*4* at 176.8 cM on chromosome 6 (Fig. 5; Supplemental Table 3). *FLS* could not be linked to this genetic map. Secondly, all detected flavonoids were re-analysed for mQTLs using the updated genetic map including the flavonoid candidate genes markers. Several flavonoids showed their strongest linkage (based on LOD score) to one or more of the flavonoid candidate gene markers (Fig. 5; Supplemental Table 5). For example, in addition to the one on chromosome 9, we identified a second naringenin chalcone mQTL linked to the *Ca*-*MYB12* marker on chromosome 1 (LOD score = 4.1). Together, these two mQTLs explain 37 % of the variation in naringenin chalcone. A strong mQTL for flavone *C*-glycosides was found on chromosome 6, having its strongest association with the *FS*-*2* gene (162 cM; LOD = 15.3), which encodes the last step in the biosynthesis of flavones. This QTL explains up to 40 % of the variation in flavone *C*-glycosides. A significant association of flavone *C*-glycosides was also found with the nearby *CHI*-*4* (176.8 cM; LOD = 7.7) marker. We are not sure, however, whether *CHI*-*4* represents a separate mQTL, since this locus overlaps with the *FS*-*2* QTL interval. Thirdly, we analysed the expression of the candidate genes in ripe fruits of the F2 individuals and subjected them to an eQTL analysis. For eleven genes (*Ca*-*MYB12*, *CHS*-*1*, *CHS*-*2, CHI*-*1, CHI*-*2*, *CHI*-*4*, *F3H*, *F3′H*-*1*, *F3′H*-*3*, *FLS* and *FS*-*2*), significant expression levels could be observed and these could be used for eQTL analysis, while three candidate genes (*CHS*-*A*, *CHI*-*3*, and *F3′H*-*2*) were not expressed in pepper fruit. Strong eQTLs were found for *CHS*-*1*, *CHS*-*2*, *CHI*-*2, FLS* and *Ca*-*MYB12*, overlapping with the position of the naringenin chalcone mQTL at the *Ca*-*MYB12* marker on chromosome 1 (Fig. 5). eQTLs for *CHS*-*1*, *CHS*-*2, CHI*-*1,* *CHI*-*2* and *FS*-2 also co-localised with the naringenin chalcone mQTL on the top of chromosome 9. In addition to the flavone *C*-glycosides, the *FS*-*2* marker on chromosome 6 was associated with an eQTL of the corresponding *FS*-*2* gene. Similarly, a *CHI*-*4* eQTL showed the strongest association with the SNP marker in the *CHI*-*4* gene. Additional eQTLs were found for *F3′H*-*1* on chromosome 3, *F3′H*-*4* on chromosome 4 and *F3H* on chromosome 8. None of these eQTLs co-localised with a flavonoid mQTL, neither did they have an association with any of the flavonoid genes mapped in this study. It should, however, be noted that these three genes were not among the set of mapped flavonoid genes.

## Discussion

The cross between *C. annuum* AC1979 (no. 19) and *C. chinense* No. 4661 Selection (no. 18) generated a varied F2 population that genetically segregated for fruit colour as well as for the composition and level of metabolites in ripe fruits. The red fruit colour of the female parent *C. annuum* AC 1979 (no. 19) is regulated by the dominant *Y* locus, encoding capsanthin–capsorubin synthase (CCS) which catalyses the production of the red carotenoids capsanthin and capsorubin from antheraxanthin and violaxanthin, respectively (Lefebvre et al. 1998; Paran and van der Knaap 2007). The male parent, *C. chinense* No. 4661 Selection (no. 18), is brown-fruited, caused by the simultaneous accumulation of both red carotenoids and green chlorophyll B. Brown-fruited pepper contain a single recessive *cl* (*chlorophyll retainer*) locus combined with the dominant *Y* locus (Efrati et al. 2005; Roca et al. 2006) and the crossing of both parents (*y* ^+^ *cl* ^+^ × *y* ^+^ *cl*) resulted in a red-fruited F1 (*y* ^+^ *y* ^+^ *cl* ^+^ *cl*). The F2 fruit colour segregated into the expected 3:1 (red:brown) ratio, due to segregation of the *cl* gene.

The crossing of the parents with different metabolite profiles revealed the segregation of metabolites detected in either or both parents over the F2 plants, as we observed for the metabolites in groups A, C and D (Fig. 3b). Based on metabolite profile, the F1 hybrid was most similar to the male parent *C. chinense* No. 4661 Selection (no. 18), since they clustered together into genotype cluster III in both the PCA (Fig. 3a) as well as the HCA (Fig. 3b). This cluster also contained the majority of F2 plants, indicating that the biochemical profile of the male parent (no. 18) was dominant over the female parent (no. 19) in both the F1 and the F2. This may suggest that the male parent (no. 18) contains many dominant alleles for metabolite production. However, we cannot exclude that several mQTLs from the female parent may be linked to lethal traits coming from the wild female parent, which may lead to lethality or reduced flower and fruit set and thus to a skewed segregation. This is supported by the fact that from 201 F2 plants, only 113 set fruit and hence could be used in this study. In addition, a skewed segregation for several markers has indeed been observed by genetic analysis of the F2 population (Maharijaya et al. 2012b). Ripe fruits from both the F1 hybrid as well as several F2 plants contained metabolites that were not detected in any of the parents (metabolite clusters B, E and F). These metabolites are likely the result of epistatic effects of alleles from both parents. In addition to such epistatic effects, we found several metabolites that were significantly (up to 12-fold) more abundant in some F2 plants compared to both parents and the F1 hybrid (particularly flavonoid glycosides in metabolite group D, such as flavone *C*-glycosides, flavonol *O*-glycosides and naringenin chalcone). This may be caused by a combination of positive alleles derived from both parents, a phenomenon called transgressive segregation. Alternatively, such extreme phenotypes may also reflect environmental variation or an interaction between genotype and environment. Replicated trials would be needed to distinguish between these different possibilities. Using the segregation in metabolite profiles we could define various mQTLs from the correlation between the genetic and metabolic data sets. At least 231 (43 %) of the 542 metabolites could be linked to one or more genetic markers. The mQTLs were unevenly distributed over the pepper genome, as we could identify mQTL hotspots on chromosome 9 showing an association with up to 103 metabolites. This suggests that large groups of metabolites share a common genetic region, encoding one or more genes that regulate the accumulation of those metabolites. For example, on the QTL hotspot at 75 cM on chromosome 9 we found co-localisation of 16 as-yet-unknown metabolites which all showed a typical doubly charged ionisation behaviour in our LCMS set-up, suggesting the presence of a regulatory gene driving the synthesis of these compounds or a structural gene at an early step in the biosynthetic pathway leading to these doubly charged compounds.

The mQTL hotspot on the bottom of chromosome 9 (99 cM) was linked to four acyclic diterpenoids, also called capsianosides (Supplemental Table 4). Accurate mass MS and MS^n^ fragmentation information revealed that these capsianosides contained different decorations of the basic diterpene skeleton (Supplemental Table 2). For example, capsianoside VIII has a diglucoside conjugated to the C-3 position of the capsianoside aglycone, whereas capsianoside IX has a triglucoside at this position (Lee et al. 2006; Supplemental Fig. 3). These five capsianosides accumulated in genotypes homozygous for the maternal allele (parent no. 19 and F2 plants) of the corresponding marker, suggesting that this allele may encode a regulatory gene which up-regulates the capsianoside biosynthetic pathway. Alternatively, it is possible that this QTL region encodes one or more modifying enzymes, such as glycosyltransferases, which lead to the production of these structurally different capsianosides.

Both parents used in this study were pungent, but showed quantitative differences in capsaicin content (Wahyuni et al. 2011). In this study we mapped a QTL for the capsaicin analogue, which was shown to be highly correlated with total capsaicin content (Wahyuni et al. 2013), on chromosome 7. In pepper, QTLs that control the capsaicinoid content in ripe fruits have been reported in a F3 population derived from an interspecific cross between the mildly pungent *C. annuum* cv. NuMex RNaky and the highly pungent *C. frutescens* accession BG2814-6 (Ben-Chaim et al. 2006). Two capsaicinoid QTLs in this F3 population were located on chromosome 2 and 7. The QTL on chromosome 2 encodes *AT3* (acyltransferase), the structural gene encoding the enzyme responsible for capsaicinoid condensation (Blum et al. 2002; Mazourek et al. 2009). Candidate genes have also been proposed for the QTL on chromosome 7, for example the putative aminotransferase gene *pAMT*, which may encode the enzyme catalysing the formation of vanillylamine from vanillin (Mazourek et al. 2009). The co-localisation of a QTL for capsaicin content on chromosome 7 in both populations may point to the same underlying candidate gene. This will be examined in a follow-up study.

Several flavonoid mQTLs were detected in this F2 population (Supplemental Table 4). To get more insight into the genes and molecular mechanisms underlying these QTLs, we (1) mapped the position of five flavonoid candidate genes and integrated these data into an updated genetic map, (2) performed an eQTL analysis based on gene expression data of 14 flavonoid genes and (3) re-analysed the flavonoid mQTL data using the updated genetic map (Fig. 5; Supplemental Table 5). This allowed the detection of a new QTL for naringenin chalcone on chromosome 1, with its QTL maximum at the *Ca*-*MYB12* gene. Expression QTLs for *Ca*-*MYB12*, *CHS*-*1*, *CHS*-*2*, *CHI*-*2* and *FLS* mapped at the same position. This is fully in line with results observed in tomato, where the *MYB12* gene has been shown to regulate the level of naringenin chalcone in the peel of ripening tomato fruits, through induction of the same set of flavonoid structural genes (Ballester et al. 2010; Adato et al. 2009). Although the mQTL for naringenin chalcone on chromosome 9 was confirmed, none of the candidate genes tested mapped within this QTL interval. However, significant eQTLs for *CHS*-*1*, *CHS*-*2*, *CHI*-*1*, *CHI*-*2* and *FS*-*2* mapped at the same position, suggesting that this naringenin chalcone mQTL is also driven by the action of an as-yet-unknown transcription factor gene, in analogy to the *MYB12* QTL on chromosome 1. On chromosome 6, the mQTLs for four flavone *C*-glycosides and the eQTL for flavone synthase (*FS*-*2*) showed their strongest association with the corresponding *FS*-*2* marker at 162.1 cM, explaining up to 40 % of the variation in these compounds. This suggests that *FS*-*2*, which catalyses the conversion of flavanones into flavones, is the gene regulating flavone *C*-glycoside levels in this material, through differences in its expression. Our current efforts are geared towards the elucidation of the causative mutations in the *FS*-*2* gene or its promoter leading to the observed gene expression differences.

In addition to these four flavone *C*-glycosides, three additional flavone *C*-glycosides were detected in metabolite group D of the HCA (Fig. 3b). These seven flavone *C*-glycosides were highly correlated with each other (*R* ^2^ > 0.6; Supplemental Table 6). Although only four of these seven flavone glycosides were significant in interval mapping, both the HCA and correlation analysis suggest that the *FS*-*2* locus regulates the formation of all flavone *C*-glycosides, as would be expected based on the function of the FS-2 enzyme. The lack of significance of the additional three flavone *C*-glycosides in interval mapping might be due to the limited population size, the presence of additional, undiscovered QTLs and/or environmental variation influencing the efficiency of QTL detection.

The CHI-4 locus at 176.8 cM on chromosome 6 showed an eQTL for the corresponding *CHI*-*4* gene. In addition this locus revealed significant associations with the flavone *C*-glycosides and *FS*-*2* gene expression. We cannot distinguish whether *CHI*-*4* represents a separate locus for flavone *C*-glycosides, since it overlaps with the *FS*-*2* QTL interval. However, the fact that the direction of the *CHI*-*4* eQTL is opposite to that of the *FS*-*2* and flavone *C*-glycoside QTLs argues against a second QTL for flavone *C*-glycosides (Supplemental Table 5). For several other flavonoid metabolite and expression QTLs, we did not observe a link to any of the flavonoid genes tested. This is mainly due to the limited number of flavonoid genes mapped in this study. Our results, however, demonstrate the power of a candidate gene approach to find the genes underlying important mQTLs, in the case of a well-studied metabolic pathway. We are convinced that genetic mapping of additional flavonoid candidate genes would uncover the genes underlying several additional flavonoid QTLs in this population.

All QTL results have been obtained from the analysis of a segregating F2 population. In such a population, all F2 individuals are unique and mortal. Despite the lack of biological replicates in such a population, reliable QTLs can be found, due to the fact that every genomic region is represented, and therefore replicated, in 75 % of the F2 individuals. Although reliable, the size of the QTL intervals in an F2 population is rather large, since only a few meioses, and hence recombinations, have taken place during its development. On the other hand, the relatively high level of heterozygosity present in an F2 population makes it possible to estimate the degree of dominance for detected QTLs (Semagn et al. 2010). Currently we are developing a recombinant inbred line (RIL; >F6 generation) population based on the same cross between *C. annuum* AC1979 (no. 19) and *C. chinense* No. 4661 Selection (no. 18). Individuals of such a population are homozygous for more than 97 %, can be propagated indefinitely through seeds and form a stable resource for future research. This material will be used to further confirm and test the robustness of selected QTLs, using biological replicates in different environmental conditions.

In summary, our results suggest that the extensive biochemical variation in pepper fruit is under the genetic control of a limited number of chromosomal regions (QTL hotspots), encoding genes that regulate the accumulation of large sets of metabolites. In the coming years we expect the release of the complete sequence of the pepper genome (Park et al. 2012) and this will aid strongly in the identification of the key genes underlying important agronomic traits. Combining genetic and biochemical datasets will help breeders to develop new pepper varieties that unite a desirable taste and nutritional profile with genetic resistance to important diseases.

## Electronic supplementary material

Below is the link to the electronic supplementary material.

Supplementary material 1 (PDF 271 kb)

Supplementary material 2 (XLSX 14 kb)

Supplementary material 3 (XLSX 654 kb)

Supplementary material 4 (XLSX 18 kb)

Supplementary material 5 (PDF 41 kb)
