# Supplementary material for: Genetic mapping of semi-polar metabolites in pepper fruits (Capsicum sp.): towards unravelling the molecular regulation of flavonoid quantitative trait loci
Source: Mol Breed. 2013 Oct 23;33(3):503–18. doi: 10.1007/s11032-013-9967-0 (PMC3918126; doi:10.1007/s11032-013-9967-0)

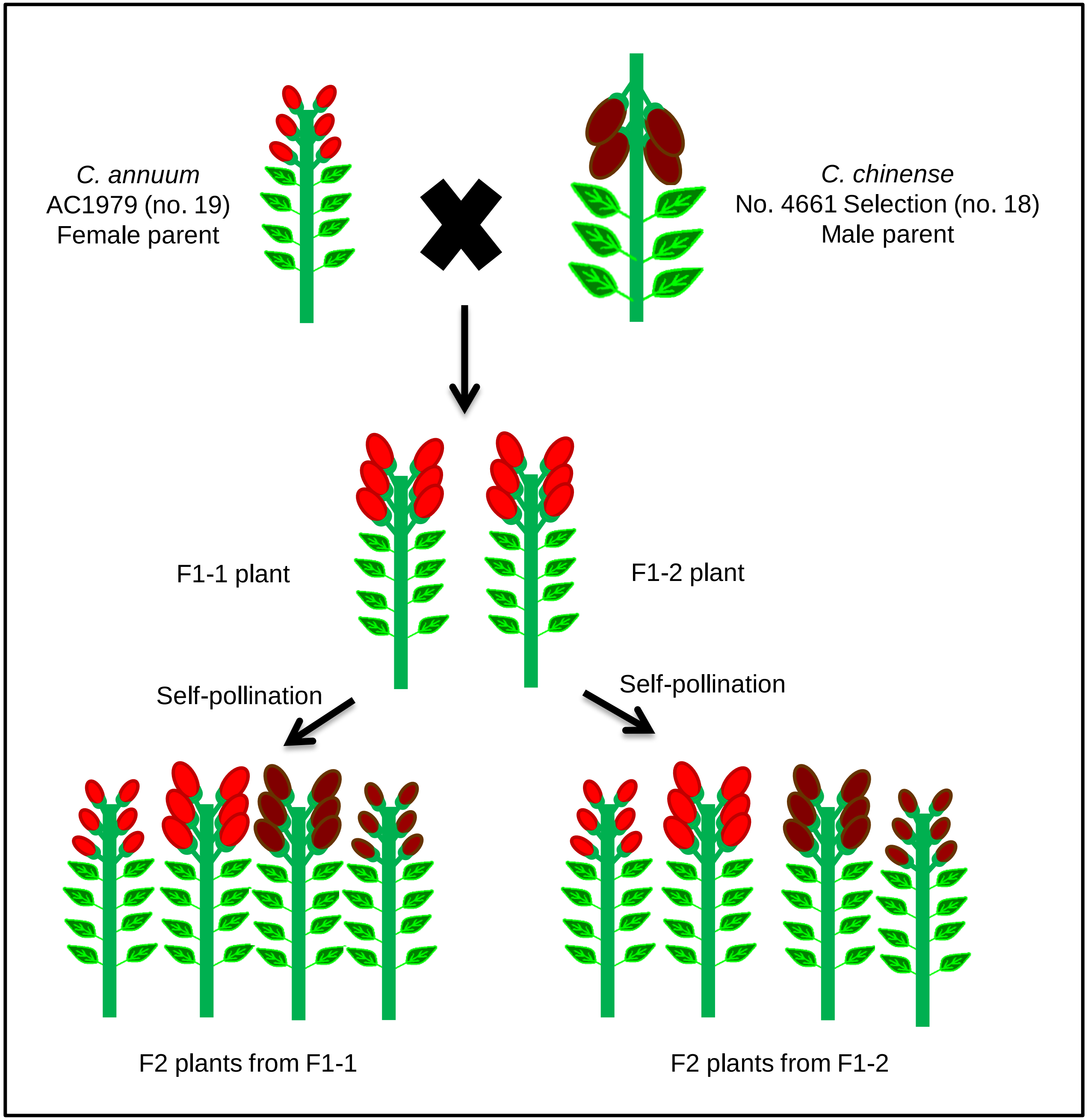

Supplemental Figure 1 A crossing scheme to develop F2 plants from *C. annuum* AC1979 (no. 19) and *C. chinense* No. 4661 Selection (no. 18). Two homogenous F1 plants were obtained by crossing two pepper accessions. Each of F1-plants was self-pollinated to produce F2 progeny. The representation of plants shown in the scheme is not in true scaled

Supplemental Figure 2 Variation in fruit morphological characters of: a, *C. annuum* AC1979 (no. 19); b, *C. chinense* No. 4661 Selection (no. 18); c, F1 plants; and d, F2 plants

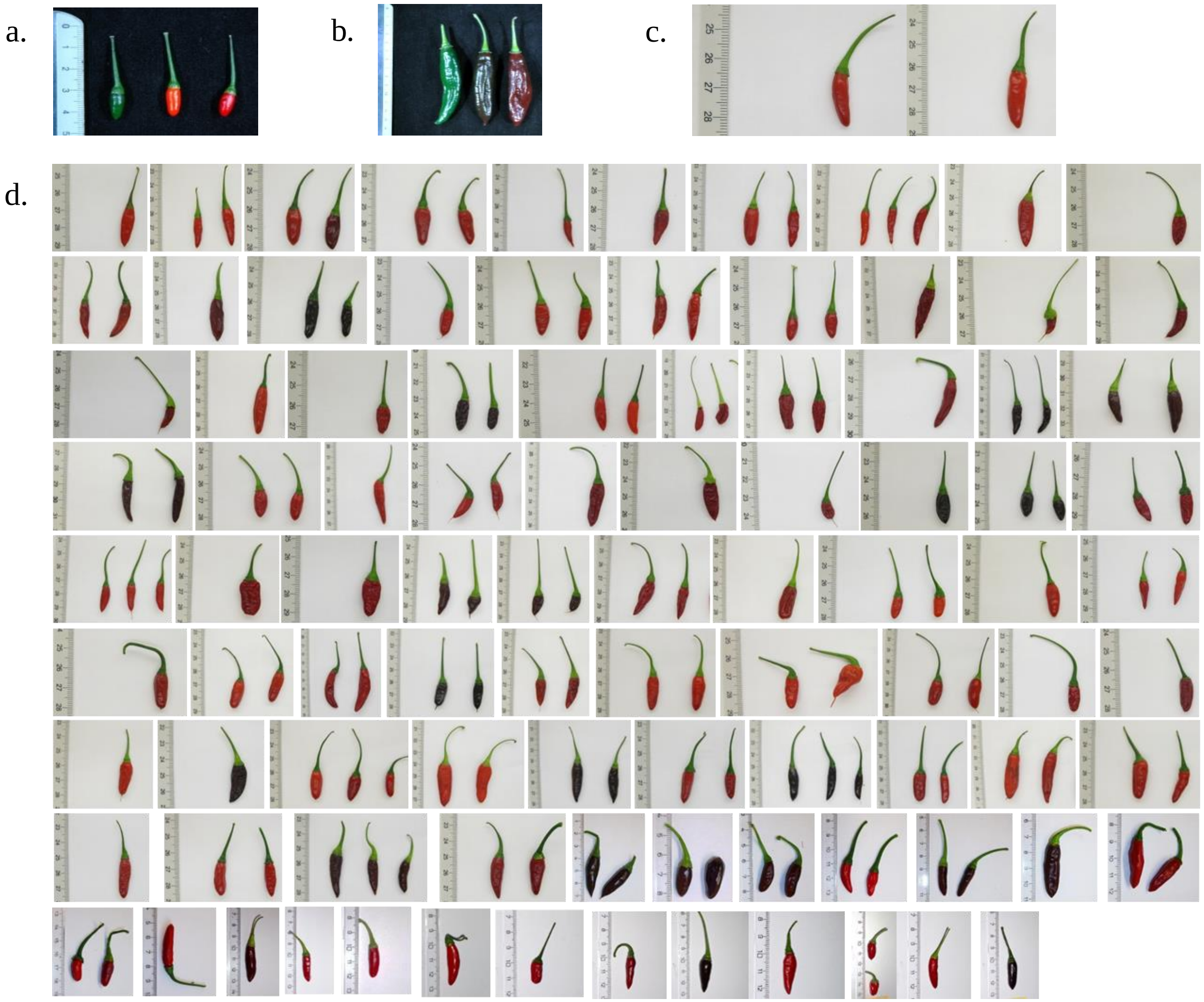

Supplemental Figure 3 MS^n fragmentation pattern of Capsianoside VIII and IX using LTQ-Orbitrap FTMS in negative ionization mode

Capsaicinoid IX

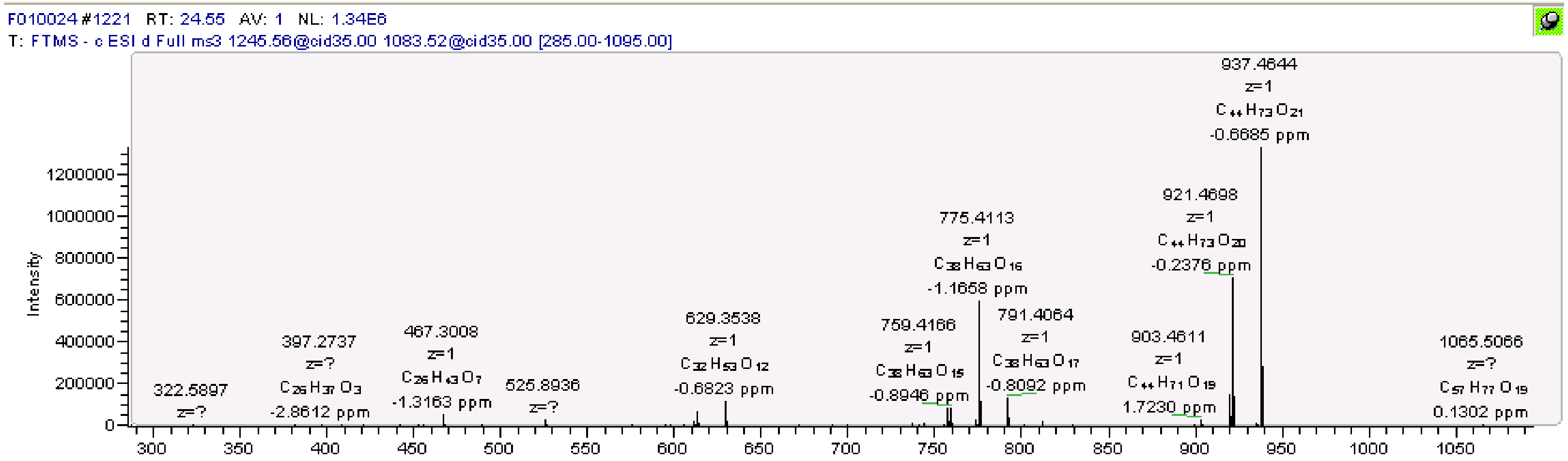

Capsaicinoid VIII

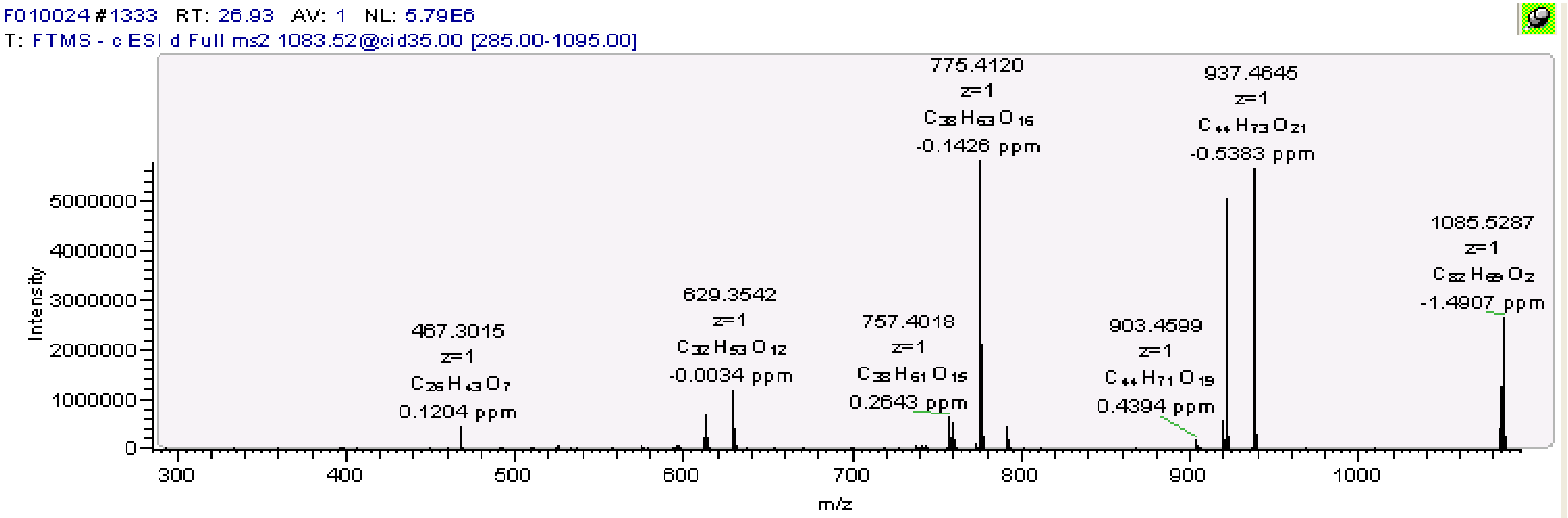

Supplement: Supplementary file 1 — Supplementary material 1 (PDF 271 kb) [file 11032_2013_9967_MOESM1_ESM.pdf]
