# Supplementary material for: Genetic mapping of semi-polar metabolites in pepper fruits (Capsicum sp.): towards unravelling the molecular regulation of flavonoid quantitative trait loci
Source: Mol Breed. 2013 Oct 23;33(3):503–18. doi: 10.1007/s11032-013-9967-0 (PMC3918126; doi:10.1007/s11032-013-9967-0)
Supplement: Supplementary file 5 — Supplementary material 5 (PDF 41 kb) [file 11032_2013_9967_MOESM5_ESM.pdf]

Supplemental Table 4 Putative metabolites associated with a quantitative trait locus

Putative metabolites	Metabolite Group[a]	Linkage Group[b]	Position	Max. LOD[c]	mu_A[d]	mu_H[e]	mu_B[f]	Closest marker
Trihydroxy-phytatetraen-one-acetyl-O-glucose	Acy. Diterp.	P02	21.00	4.4	5.29	6.85	5.11	E38M49-457
Quercetin rhamnoside-glucoside	Flav.	P02	143.72	4.2	5.51	7.22	7.00	P17M39-130
3-Methyl-1-butanol; O-[?-D-Glucopyranosyl-(1?6)-?-D-glucopyranoside]	Ile/leu deriv.	P03c	12.51	3.9	8.20	7.17	6.27	P17M32-133
Flavanone tri-methyl glucose pentose	Flav.	P06	79.86	7.8	5.21	5.20	5.84	Epms_376
Icariside E5	Lignan	P06	141.09	4.2	8.59	9.39	8.18	P11M48-159
Apigenin 6-C-pentoside-8-C-hexoside	Flav.	P06	161.35	5.6	6.68	8.74	8.77	HpmsE088-6b
Apigenin 6,8-di-C-hexoside	Flav.	P06	162.35	8.7	6.97	9.19	9.20	HpmsE088-6b
Luteolin 8-C-hexoside	Flav.	P06	165.35	3.8	6.28	8.13	7.95	HpmsE088-6b
Luteolin 6-C-hexoside	Flav.	P06	167.35	6.9	7.29	9.42	9.30	HpmsE088-6b
capsaicin/capsaicin analogue	Phenylprop.	P07	97.03	3.7	9.88	9.68	9.12	Hpms1-227_Q1
Flavanone tri-methyl glucose pentose	Flav.	P07	101.03	6.1	7.02	5.12	5.41	P17M39-199
Naringenin chalcone	Flav.	P09	13.71	6.7	7.96	8.72	9.86	Hpms1-3_Q1
Luteolin-methyl-acetyl-apiofuranosyl-hexose	Flav.	P09	67.86	4.1	5.93	5.28	5.22	P14M41-408
Capsianoside V	Acy. Diterp.	P09	90.57	6.4	5.84	10.04	10.47	P11M48-517
Capsianoside IX	Acy. Diterp.	P09	93.57	4.2	9.22	7.38	6.15	P11M48-517
Capsicoside	Saponin	P09	94.57	3.7	7.04	5.26	5.21	P11M48-517
Luteolin-methyl-O-di-hexose	Flav.	P09	96.57	6.5	9.95	6.53	5.95	P11M48-517
Capsianoside X-1	Acy. Diterp.	P09	97.57	4.3	10.27	6.45	6.89	P11M48-517
Capsianoside VIII	Acy. Diterp.	P09	98.57	4.6	11.40	7.53	6.97	P11M48-517
Quercetin 3-O-rhamnoside	Flav.	P10b	7.00	4.0	6.28	8.31	8.24	E38M49-534
Ferulic acid-hexose II	Phenylprop.	P11	23.68	4.3	5.90	6.77	7.01	Epms_561-11_Q1
Icariside E5	Lignan	P11	42.12	6.0	8.08	8.91	9.51	Epms_391-11_Q1
Capsianoside IV	Acy. Diterp.	P12b	38.15	3.9	8.72	6.67	5.40	E32M49-597

[a] Metabolites were grouped into flavonoids (Flav.), acyclic diterpenoids (Acy. Diterp.), phenylpropanoids (Phenylprop.), ile/leu derivatives (Ile/leu deriv.), fatty acid derivative (FA deriv.), lignan and saponin

[b] Linkage group was annotated by the chromosome number after the alphabet P and followed by the small alphabet. Linkage map was constructed with AFLP and microsatellite markers as described by Maharijaya et al. (2012b).

[c] LOD = logarithm of odds corresponds to a genome wide confidence level. LOD corresponds to the maximum LOD score.

[d] mu_A = trait means of AA homozygous P1, *C. annuum* AC1979 (no. 19)

[e] mu_H = trait means of AB heterozygous progeny

[f] mu_B = trait means of BB homozygous P2, *C. chinense* No. 4661 Selection (no. 18)

Supplemental Table 5 Flavonoid QTLs and flavonoid candidate gene expression QTLs in pepper

Linkage Group[a]	Gene transcript/ Metabolite	Position	LOD[b]	mu_A[c]	mu_H[d]	mu_B[e]	Locus
P01	*Ca-MYB12*	89.63	21.9	7.56	10.68	12.47	Ca-MYB12
	CHS-1	89.63	9.6	13.05	15.24	16.53	Ca-MYB12
	CHS-2	89.63	9.9	13.15	15.39	16.57	Ca-MYB12
	CHI-2	89.63	4.6	9.99	10.65	12.35	Ca-MYB12
	FLS	89.63	3.6	6.22	7.18	7.95	Ca-MYB12
	Naringenin Chalcone	89.63	4.1	7.87	8.99	9.38	Ca-MYB12
P03c	*F3'H-1*	40.38	9.4	12.60	15.40	16.21	Epms_386-3_Q1
P04a	*F3'H-3*	84.17	7.61	3.19	5.70	5.97	P11M48-220
P06	*FS-2*	162.13	7.8	7.79	9.08	11.08	FS-2
	Apigenin 6-*C*-pentoside-8-*C*-hexoside	162.13	9.0	6.63	8.84	8.83	FS-2
	Luteolin 6-*C*-hexose	162.13	13.9	6.78	9.64	9.54	FS-2
	Luteolin 8-*C*-hexose	162.13	8.4	5.82	8.28	8.26	FS-2
	Apigenin 6,8-di-*C*-hexoside	162.13	15.3	6.86	9.28	9.33	FS-2
	CHI-4	176.82	33.0	13.13	11.67	6.15	CHI-4
	FS-2	176.82	4.5	8.20	9.03	10.48	CHI-4
	Apigenin 6-*C*-pentoside-8-*C*-hexoside	176.82	5.0	7.21	8.61	8.81	CHI-4
	Apigenin 6,8-di-*C*-hexoside	176.82	7.7	7.53	8.98	9.31	CHI-4
	Luteolin 6-*C*-hexose	176.82	6.1	7.62	9.32	9.47	CHI-4
	Luteolin 8-*C*-hexose	176.82	3.6	6.59	8.12	8.04	CHI-4
P08	*F3H*	23.72	3.9	11.90	12.45	13.29	E38M49-585
P09	*CHS-1*	15.08	5.8	13.99	14.56	16.34	E36M48-392
	CHS-2	15.08	5.7	14.11	14.71	16.42	E36M48-392
	CHI-1	15.08	3.6	6.28	7.10	8.41	E36M48-392
	CHI-2	15.08	4.2	10.31	10.35	11.75	E36M48-392
	FS-2	15.08	3.6	8.60	9.12	10.65	E36M48-392
	Naringenin chalcone	15.08	6.6	8.00	8.68	9.77	E36M48-392
	Luteolin methyl acetyl apiofuranosyl hexose	67.86	4.1	5.93	5.28	5.22	P14M41-408
	Luteolin methyl-*O*-di-hexose	96.57	6.5	9.95	6.53	5.95	P11M48-517

[a] Linkage group was annotated by the chromosome number after the alphabet P and followed by the small alphabet. Linkage map was constructed with AFLP and microsatellite markers as described by Maharijaya et al. (2012b).
[b] LOD = logarithm of odds corresponds to a genome wide confidence level. LOD corresponds to the maximum score at the position of marker
[c] mu_A = trait means of AA homozygous P1, *C. annuum* AC1979 (no. 19)
[d] mu_H = trait means of AB heterozygous progeny
[e] mu_B = trait means of BB homozygous P2, *C. chinense* No. 4661 Selection (no. 18)
The grey boxes indicate the parent of which the trait was derived from

Supplemental Table 6 Pearson correlation analysis of flavone *C*-glycosides

HCA Order	HCA Group	Putative ID	Correlation value to Apigenin 6,8-di-*C*-hexoside (R^2)
249	D	Apigenin 6,8-di-*C*-hexoside	1
242	D	Apigenin 6-*C*-pentoside-8-*C*-hexoside	0.9
244	D	Luteolin 6,8-di-*C*-hexoside	0.7
245	D	Luteolin 6-*C*-hexoside-8-*C*-pentoside	0.6
246	D	Luteolin 6-*C*-pentoside-8-*C*-hexoside	0.6
248	D	Luteolin 8-*C*-hexoside	0.8
250	D	Luteolin 6-*C*-hexoside	0.9
